# Global research trends and focus on the link between neutrophil extracellular traps and tumor: a bibliometric and visualization analysis from 2006 to 2024

**DOI:** 10.3389/fimmu.2024.1452104

**Published:** 2024-09-24

**Authors:** Chaoyue Xiao, Xiang Feng, Zengyi Zhao, Gouping Ding, Yawen Gao

**Affiliations:** Department of Oncology, The Second Xiangya Hospital, Central South University, Changsha, China

**Keywords:** neutrophil extracellular traps, tumor, tumor microenvironment, immunotherapy, bibliometrics, VOSviewer, CiteSpace, R-bibliometrix

## Abstract

**Background:**

Neutrophil extracellular traps (NETs) have long been consistently considered an innate immune defense against foreign pathogens, but this oversimplified view has decelerated the progression of perceiving NET biology in chronic diseases. It is now increasingly accepted that NETs are not exclusive to anti-infection responses, but are also central players with a double-edged sword role in cancer progression. NETs have gradually emerged as tumor diagnostic, predictive, and prognostic biomarkers, and strenuous endeavors have been devoted to tapping their potential as new therapeutic targets. Correspondingly, the boom in studies on NETs and tumors in recent years has achieved a series of scientific outputs, which opens up a new perspective for perceiving the sophisticated landscapes of the tumor immune microenvironment. However, there is still much room to translate NET-targeted immunotherapies into clinical practice. Therefore, it is necessary to explore the knowledge structure and latent hotspots of the links between NETs and tumors using bibliometric analysis.

**Methods:**

NETs and tumor publications from 2006 to 2024 were extracted from the Web of Science Core Collection. Bibliometric analysis and visualization were conducted using Microsoft Excel, VOSviewer, CiteSpace, and R-bibliometrix.

**Results:**

The analysis included 1,339 publications authored by 7,747 scholars affiliated with 1,926 institutions across 70 countries/regions with relevant articles published in 538 journals. Despite China’s maximum number of publications, the United States has continued to dominate the field as a global cooperation center with overwhelming citation counts. Frontiers in Immunology published the most number of publications, whereas Blood was the most cited journal. Wagner, Denisa D. and Kaplan, Mariana J. are concurrently in both the top 10 most prolific authors and cited author lists. Tumor microenvironment and immunotherapy will likely be the focus of future research.

**Conclusions:**

A comprehensive bibliometric analysis was first conducted to map the current landscape and knowledge structure of the link between NETs and tumors in the hope of providing guidance and fresh perspectives for further research in this field. NETs are promising antitumor targets, and perhaps the eventual destination in the realm is to translate NET-targeted immunotherapies into clinical practice.

## 1 Introduction

Comprehending tumor immunology is a progressive undertaking, with an earlier focus chiefly centered on adaptive immunity. Nevertheless, merely targeting the adaptive immunocytes reaped gloomy efficacy in knocking down the ramparts of tumor-induced immunosuppression. This limitation highlights the significance of the broader tumor microenvironment (TME), where innate immunity plays a vital role in tumor immune surveillance and evasion. As perceptions of the TME have grown more profound, attention has shifted from adaptive immunity to innate immunity. As a fundamental element of innate immunity, neutrophils are dominant in inflammation, notably in acute inflammation. Their ephemeral lifespan and inability to proliferate delimit them from other immunocytes and once skewed people to depreciate their biology in tumors, which are chronic malignant diseases (1). Neutrophils are pivotal innate immune signaling hubs that transmit or activate mass pathways imperative for adaptive immunity, and there has recently been a surge of interest in exploring neutrophils and tumors (2).

In 2004, Brinkmann et al. discovered that neutrophils undergo a peculiar death modality known as NETosis, wherein they usually extrude a chromatin backbone coated with specific nuclear and cytoplasmic proteins that form web-like filamentous extracellular structures called neutrophil extracellular traps (NETs) to capture and kill bacteria (3). NETs have long been consistently judged as an innate immune defense against foreign pathogens, but this oversimplified view of NETs has decelerated the perception of their biology in chronic diseases, encompassing tumors. In the past decade, the role of NETs in tumor immunity have been drawing more attention, and it is now increasingly accepted that NETs are not exclusive to anti-infection responses but are also central players with multifaceted effects on all stages of cancer progression (4–7). NETs have gradually emerged as tumor diagnostic, predictive, and prognostic biomarkers, and strenuous endeavors have been devoted to tapping their potential as new therapeutic targets (8–12). Correspondingly, the boom of studies on NETs and tumor in recent years has achieved a series of scientific outputs, which opens up a new perspective for perceiving the sophisticated landscapes of tumor immune microenvironment. Yet it remains much left to translate NET-targeted immunotherapies into clinical practice. Therefore, it is necessary to comprehensively appraise the research status, focal point, and evolutionary trend of the link between NETs and tumors.

Bibliometrics is an emerging interdisciplinary science that delivers a comprehensive and objective evaluation of knowledge carriers by mathematics and statistics, wherein bibliographic analysis fosters scholars to digest the progression of specific topics and unmasks the evolution trend of this field. Nonetheless, bibliometric analysis of NETs and tumors remains a challenge. Hence, this study aimed to map the current landscape and knowledge structure of the link between NETs and tumors in the hope of providing fresh clues and ideas for future research.

## 2 Materials and methods

### 2.1 Data source and search strategy

**Figure 1:**
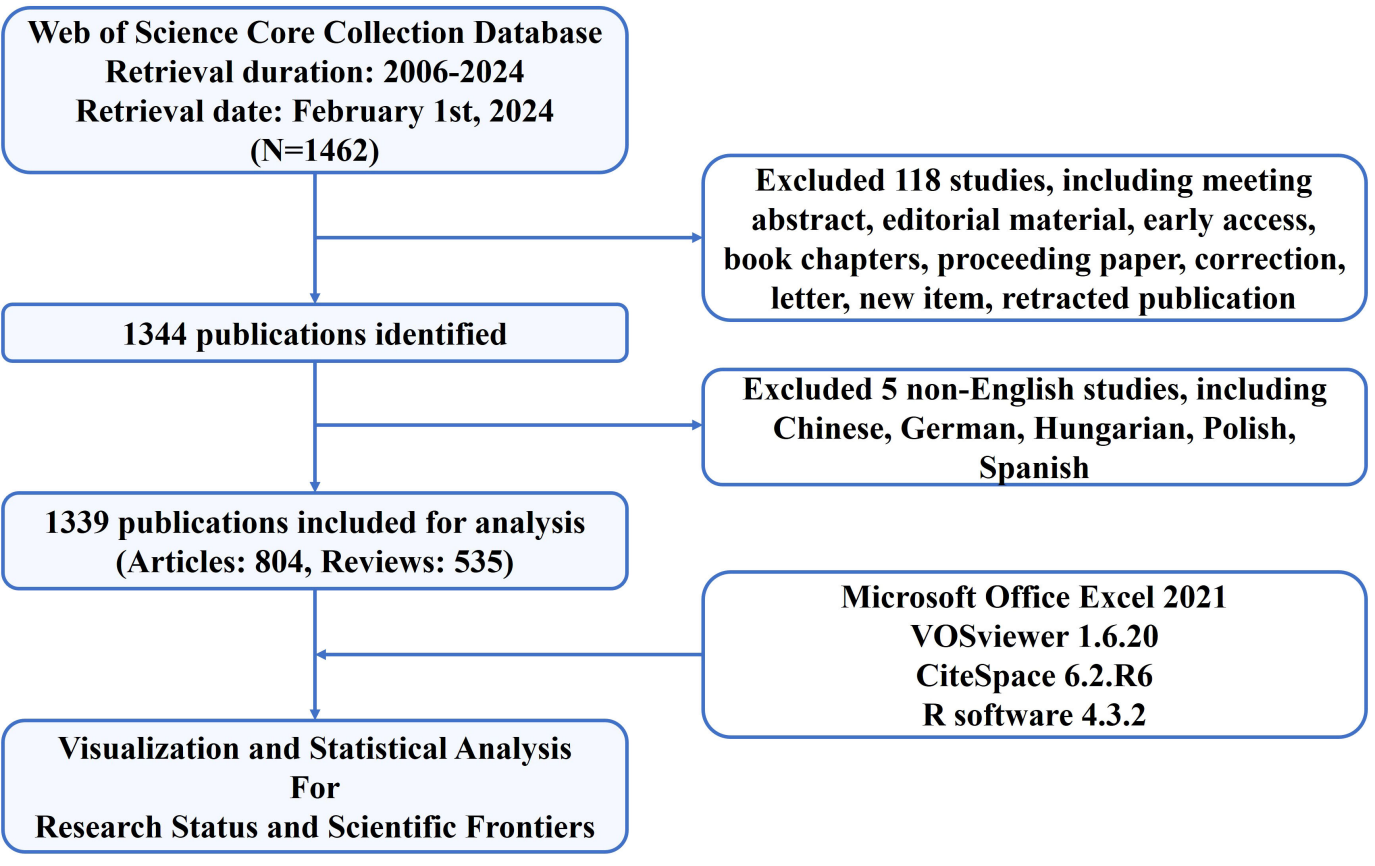
Flowchart of the screening process.

The data for bibliometric analysis of this study were extracted from the Web of Science Core Collection (WoSCC) database, which is accessible at: https://www.webofscience.com/wos/woscc/basic-search. To avoid data bias and ensure consistency in our data, two reviewers separately searched original articles and reviews from 2006 to 2024 and downloaded all relevant information in plain text format in a single day (2024.02.01). The search formula was set as follows: TS = (“neutrophil*”) AND (“NETs” OR “neutrophil extracellular trap*” OR “NETosis”) AND (“tumor*” OR “tumour*” OR “cancer*” OR “neoplasia*” OR “neoplasm*” OR “malignant*’” OR “carcinoma*” OR “adenocarcinoma*” OR “sarcoma*” OR “oncolog*” OR “lymphoma*” OR “leukemia*” OR “leukaemia*” OR “melanoma*”). Among the diverse forms of relevant publications, only English articles and reviews were included and analyzed. Finally, 804 articles and 535 reviews were included. A study flowchart for the inclusion and exclusion of publications is shown in **Figure 1**.

### 2.2 Visualization and statistical analysis

A total of five scientific tools were used to perform bibliometric and visual analyses in the study. Microsoft Office Excel 2021 was used to construct a polynomial regression model for predicting the number of publications in 2024. VOSviewer (Version 1.6.20) is a widely used bibliometric visualization software that was utilized to conduct a co-authorship analysis of country/author/institution, co-citation analysis of journals, and keyword co-occurrence analysis. CiteSpace (version 6.2.R6) is another mainstream bibliometric tool that was mainly employed to visualize the co-citation network for identifying key references and the strongest citation bursts of references and keywords in our study. In addition, CiteSpace has created a dual-map overlay of journals. The R software (version 4.3.2) is a language and environment tool that is extensively used for statistical computing and graphics. In this study, the Bibliometrix package 3.2.1 in R and two online platforms (https://bibliometric.com/ and https://flourish.studio/examples/) were applied to present the global cooperation atlas, authors’ production over time, document citation analysis, and trend topics, among others.

## 3 Results

### 3.1 Research profile

**Figure 2:**
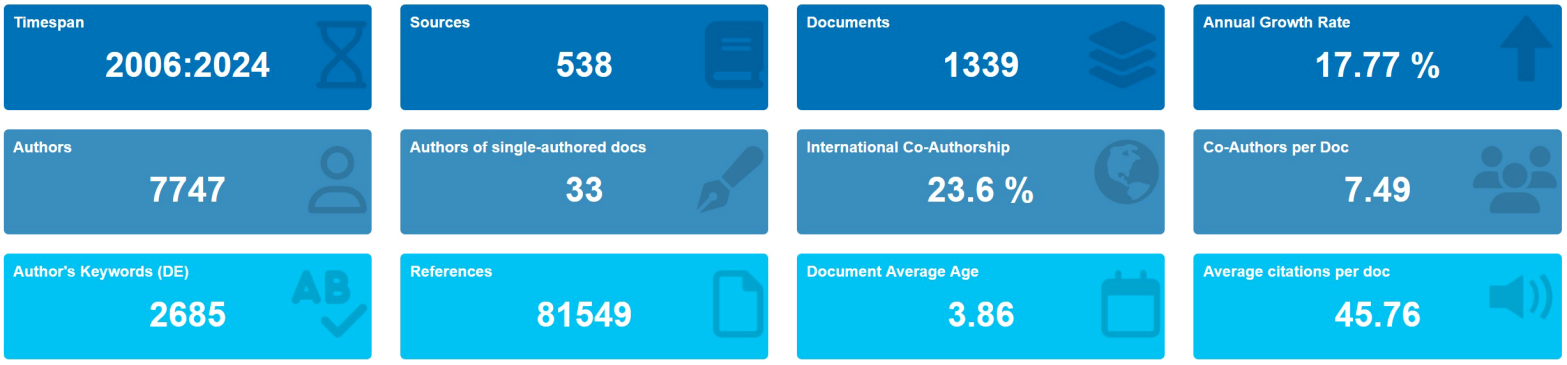
Basic information on 1,339 relevant publications included. The period of included articles, number of journal categories, total number of articles, annual growth rate, total number of authors, number of articles published by a single author, proportion of international co-authors, number of co-authors of an article, keywords given by the author, number of references cited, average life span of each article, and average number of citations per article.

According to the screening process (**Figure 1**), 1,339 eligible publications, including 804 articles and 535 reviews published from 2006 to 2024, were included. The current status is presented using the Bibliometrix package in **Figure 2**. The study included 1,339 publications from 538 journals with an annual publication growth rate of 17.77%. There were 7,747 authors, and a single author wrote 33 articles. Authors with international cooperation accounted for 23.6% of the total sample. Each publication had an average of 7-8 authors; 2,685 author keywords were provided, and 81,549 references were cited. The average life span of each publication from being noticed to being unknown is 3.86 years; each publication has been cited an average of 45–46 times. The H-index for all the publications was 113.

### 3.2 The annual trend of global publication quantity

**Figure 3:**
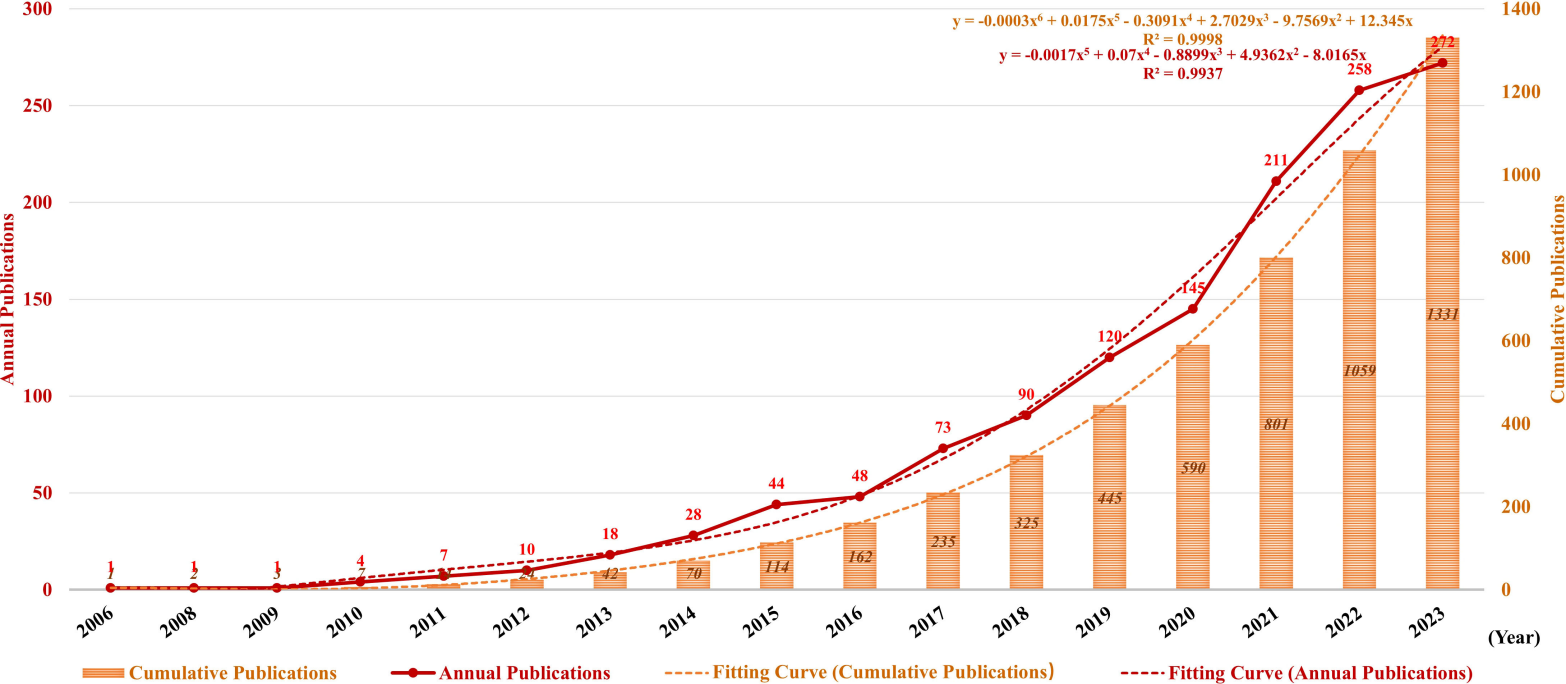
Annual and cumulative growth trends in publications. The red and orange dotted lines represent the trend-fitted curves obtained using the polynomial regression model. The correlation coefficients (R^2^) are displayed in the figure.

**Figure 3** illustrates the publication trend since its inception, which manifests a significant and steady surge in the annual volume of publications in this field. The publication rate has remained high, with more than 100 publications per year since 2019. Since 2020, 887 publications have been published, accounting for 66.24% of the total in the past 20 years. Additionally, the polynomial curve demonstrated a statistically significant positive correlation between the quantity of annual/cumulative publications and the year of publication (R^2^ = 0.9998 and R^2^ = 0.9937, respectively). According to the fitting curve, the number of annual/cumulative publications in 2024 will be 401 and 3,240, respectively. These figures indicate that NETs are becoming an emerging focus of tumor research and relevant theories are booming.

### 3.3 Country/region and institution analysis

**Figure 4:**
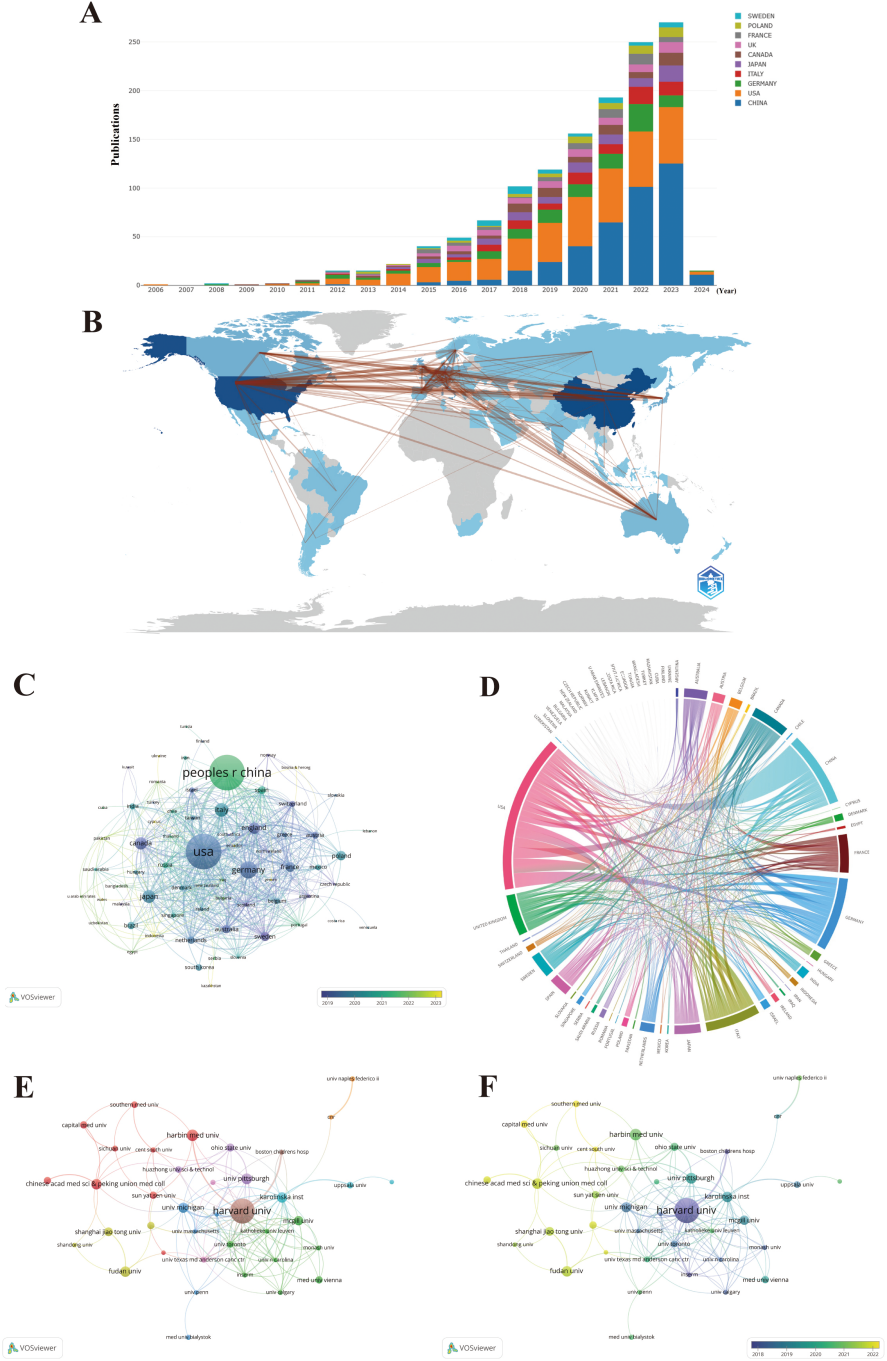
Maps of country/region and institutional analysis. **(A)** Trends in the annual number of publications from the top 10 countries/regions. **(B)** Geographic distribution of publications across different countries/regions and their international collaborations. **(C)** Visualization of international collaboration between countries/regions. **(D)** Overlay visualization of country/region co-authorship. **(E)** Co-authorship networks among institutions. **(F)** Overlay visualization of institutional co-authorship.

A total of 70 countries/regions have contributed to the literature in this field, with China (383 publications, 28.60%), the United States (381 publications, 28.45%), and Germany (119 publications, 8.89%) publishing the most (**Figures 4A, B**; **Table 1**). These three countries contributed to approximately 65.94% of the total publications on this topic from 2006 to 2024. Notably, the publication count for most countries/regions and institutions is not proportional to the citation count in this field. Despite producing more publishments than the United States, China has less than half the citations of the United States, partially because China has recently cut a striking figure as an emerging country on this topic, as shown in **Figure 4C**. **Figure 4C** shows an overlay visualization of country/region co-authorship, wherein 68 countries/regions form mutual connections. The interactive cooperation map plots most communication and collaboration confined to Europe, North America, and a handful of Asian countries (**Figures 4B–D**). Links between countries/regions represent cooperative relationships, with thicker lines denoting stronger collaboration. The countries with the highest number of publications, such as China, the United States, Germany, Italy, and Japan, possess notably close partnerships with other countries and regions. In particular, the United States was at the center of the cooperation map (**Figure 4C**).

**Table 1:** The top 10 countries/regions and institutions ranked by publications and citations.

Rank	Country	Publications	Country	Citations	Institution	Publications	Institution	Citations
1	CHINA	383	USA	29,131	Harvard University	55	Harvard University	11,899
2	USA	381	CHINA	13,457	Harbin Medical University	25	University of Michigan	6,993
3	GERMANY	119	GERMANY	11,474	University of Pittsburgh	24	Karolinska Institute	6,472
4	ITALY	82	ENGLAND	10,332	Karolinska Institute	23	Institut national de la santé et de la recherche médicale	5,974
5	JAPAN	76	ITALY	9,045	Fudan University	23	University of Massachusetts	5,392
6	CANADA	68	SWEDEN	8,835	Chinese Academy of Medical Sciences & Peking Union Medical College	23	University of Pittsburgh	4,872
7	ENGLAND	59	CANADA	8,686	University of Michigan	22	Massachusetts General Hospital	4,704
8	FRANCE	49	FRANCE	8,466	McGill University	21	The University of Texas MD Anderson Cancer Center	4,072
9	POLAND	45	AUSTRIA	7,245	Shanghai Jiao Tong University	19	University of Toronto	4,044
10	SWEDEN	44	AUSTRALIA	6,431	The Ohio State University	18	Katholieke Universiteit Leuven	4,034

The distribution of institutions is in accordance with countries/regions, based on geographical location. For instance, China has emerged as the leading global producer of publications in the field, transcending all other countries/regions, and four out of the top 10 most productive institutions are also held by China. Significantly, Harvard University overwhelmingly held the top rank, with the highest number of publications and citations (**Table 1**). To further discover the status of inter-agency cooperation, 1,339 publications were imported into VOSviewer, where the parameter was set as an institution to publish at least 10 publications. Among the 1,926 institutions, 38 met the inclusion thresholds and were connected to each other (**Figure 4E**). In **Figure 4E**, the size of the circle represents the number of publications, and divergent colors denote divergent cooperative clusters, which indicate cooperation in which the intensity of the cooperation between institutions is denoted by the width of the lines joining them. The 38 institutions exhibit close link strengths and are classified into nine clusters according to the degree of collaboration. Specifically, Harvard University (N = 66), Karolinska Institute (N = 40), and University of Michigan (N = 26) possessed the highest total link strength, indicating active collaboration with other institutions in this field. When combined with the overlay visualization map of the institution co-authorship analysis (**Figure 4F**), it can be observed that the above three institutions in the blue cluster are considered predecessors. Many Chinese institutions are located in the yellow cluster, denoting that they have initially set foot in this field in recent years. This result is in line with **Figure 4C**, which at least partially explains why the above three institutions possess the highest total link strength and citations, while Chinese institutions lag behind in these aspects. Harvard University is the most senior institution in the field, given its highest number of publications, citations, intensity of collaboration, and history of development. Nevertheless, given the hyperproductivity of publications, China has great potential in this field. Especially note that emerging Chinese institutions should strengthen cooperation with senior occidental institutions in the field given Chinese institutions gather in the left corner of **Figures 4E, F**.

### 3.4 Author and co-cited author analysis

**Figure 5:**
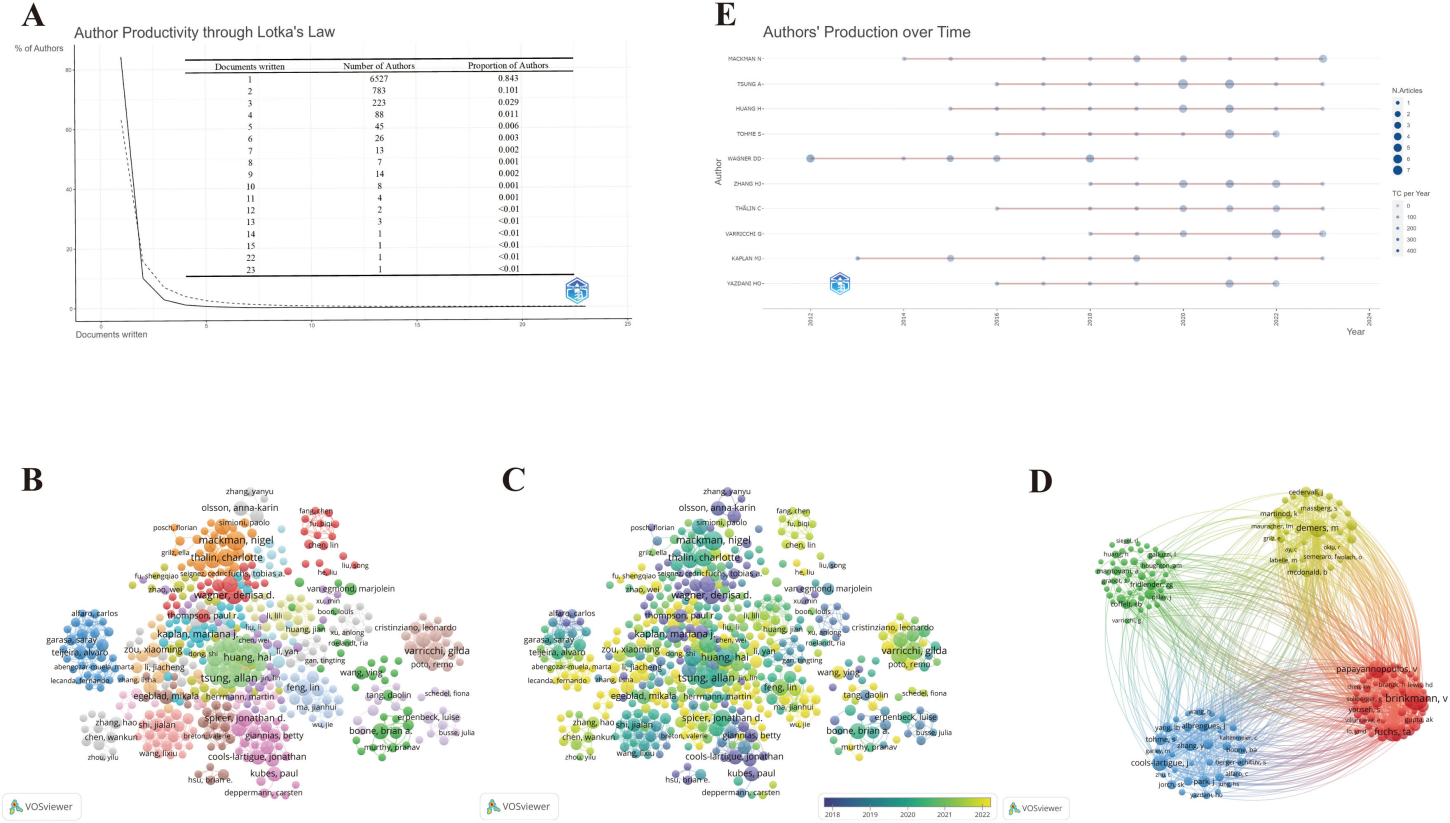
Maps of author’s analysis. **(A)** Productivity of authors based on Lotka’s law. **(B)** Co-authorship network among authors. **(C)** Overlay visualization of author co-authorship. **(D)** Co-citation network among authors. **(E)** The world’s publishing career of the top 10 researchers.

A total of 7,747 authors were involved in the study of NETs and tumors, wherein 84.3% of the authors contributed solely to a single publication according to scientific productivity based on Lotka’s law (**Figure 5A**). Meanwhile, we utilized the Bibliometrix package in R to explore the authors’ local impact, and the local H-index in **Table 2** attempts to assess authors based on publications in this field. The 492 authors with co-authorship and at least two publications are shown in **Figure 5B**. Lines between circles represent cooperation, with authors in the same color cluster manifesting stronger co-authorship. This formed 24 clusters, indicating that there are many closely collaborating academic groups in the field of NETs and tumors. Nevertheless, close cooperation and communication among distinct clusters are lacking. According to the overlay visualization map of author co-authorship analysis (**Figure 5C**), authors in the blue cluster are considered pioneers in this field, whereas authors in the yellow clusters have begun to publish papers in recent years. The authors in the blue clusters have larger nodes and more connections, which means they have more publications and collaborations. Furthermore, a co-citation analysis based on the association strength algorithm was conducted to illustrate the functional and thematic impacts of 175 interconnected authors who have been cited more than 50 times (**Figure 5D**). Each node in the graph represents an author. Node size is positively correlated with the number of publication citations by the author, and the number and thickness of the lines connecting the nodes indicate the co-citation relationship between the authors. Authors denoted by the same color belong to the same cluster, suggesting that their work is frequently cited together. Apparently, the 175 interconnected authors are classified into four clusters, in which Brinkmann, Fuchs, and Papayannopoulos are represented by the largest nodes as the top three co-cited authors in this analysis, demonstrating their significant influence on the realm and their crucial role in pushing the field’s ongoing development.

**Table 2:** The top 10 authors ranked by publications and citations.

Rank	Author	Documents	Citations	H-index	Author	Citations	Documents
1	Tsung, Allan	15	1,243	13	Tang, Daolin	3,479	4
2	Mackman, Nigel	12	780	10	Wang, Ying	3,267	6
3	Huang, Hai	11	1,163	11	Wagner, Denisa D.	2,543	10
4	Tohme, Samer	11	990	10	Kubes, Paul	1,982	7
5	Wagner, Denisa D.	10	2,543	12	Wang, Yanming	1,831	7
6	Zhang, Hongji	10	727	9	Martinod, Kimberly	1,626	8
7	Thalin, Charlotte	10	652	8	Cools-Lartigue, Jonathan	1,425	7
8	Varricchi, Gilda	10	389	7	Egeblad, Mikala	1,399	7
9	Kaplan, Mariana J.	9	1,383	9	Kaplan, Mariana J.	1,383	10
10	Yazdani, Hamza O.	9	947	8	Giannias, Betty	1,261	6

To further explore the top 10 scholars with the highest output, we utilized the Bibliometrix package in R to create the time graph, which portrays the number of publications each year and the total citations per year from 2006 to 2024 (**Figure 5E**), through which the careers of the scholars can be seen directly. Circle size represents the number of publications, with larger circles denoting more publications, and circle shade indicates total citations for that year, with darker colors representing more citations. The number of publications and total citations are the fundamental metrics adopted to gauge the productivity of core authors. Studies on the relationship between NETs and tumors have increased in the past decade, and an increasing number of scholars have begun to embark on this theme. Specifically, it is straightforward to deduce that Wagner’s scientific research output was distributed discontinuously from 2012 to 2019, whereas the other nine most prolific authors had come to prominence in the past decade. In particular, Mackman’s scientific research output has been ongoing since 2014, reflecting his passion and dedication to this field.

### 3.5 Journal and co-cited journal analysis

**Figure 6:**
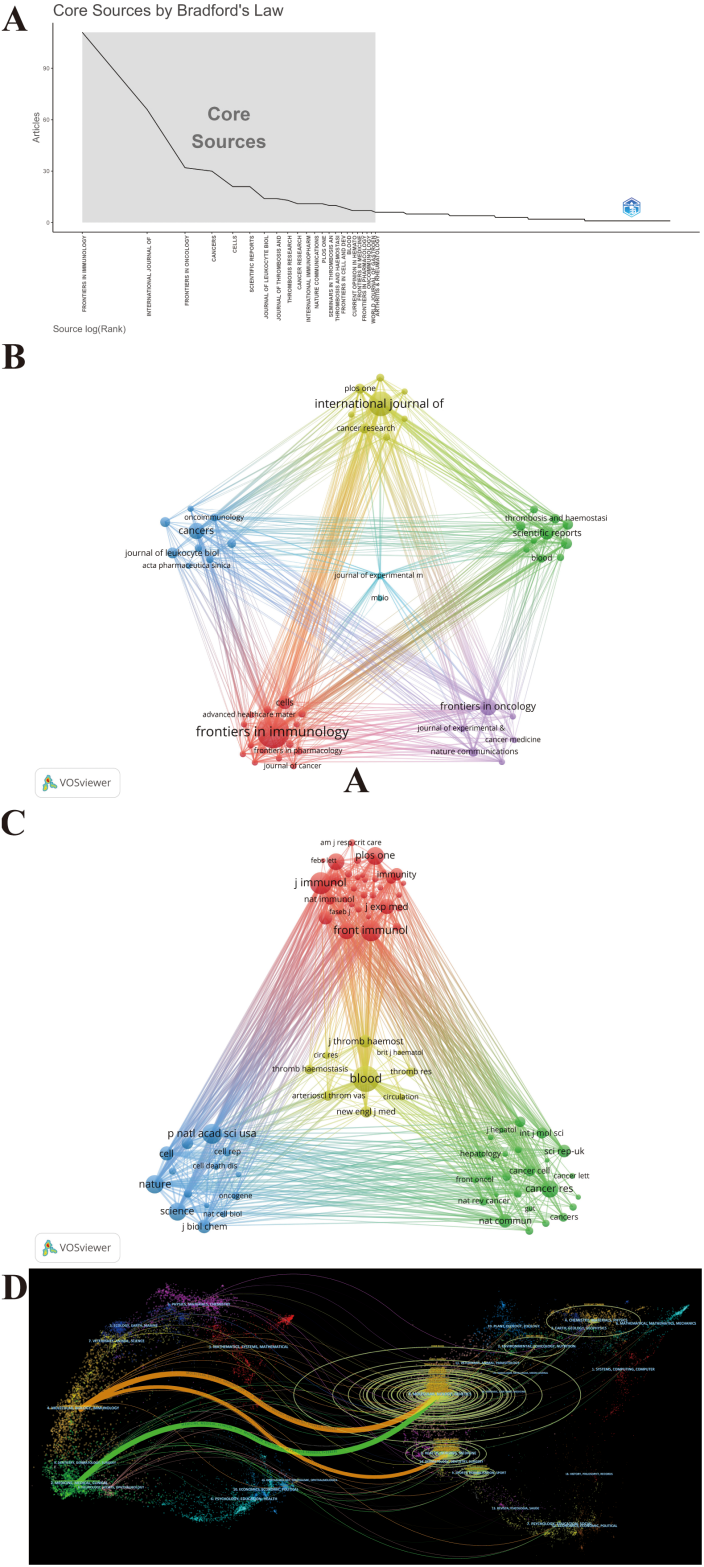
Maps of journal analysis. **(A)** 23 core journals based on Bradford’s law. **(B)** Citing network among journals. **(C)** Co-cited networks among journals. **(D)** Dual-map overlay of journals.

These publications were sourced from 538 journals. Notably, 23 journals were identified as core journals using Bradford’s Law (**Figure 6A**), comprising 444 publications. The 10 journals with the most publications in this area, along with local H-index and impact factor (IF) as indicators of impact are listed in **Table 3**. A total of 331 publications are covered in the top 10 prolific journals, representing 25.08% of all publications. These journals are more likely to accept articles on NETs. Among the top 10 prolific journals, four journals had an IF greater than 5, and six were at Q1 based on the 2023 Journal Citation Reports (JCR). Cancer Research showed the highest IF of 12.5, followed by Frontiers in Immunology at 5.7. We further filtered out 50 journals with a minimum of five relevant publications to form six clusters, thus creating a citing network diagram among journals, as shown in **Figure 6B**, where larger nodes denote a greater number of relevant publications within that journal, and the connecting lines between nodes indicate a cross-citation relationship between two journals. Obviously, the journals in which research results on NETs and tumors have been published have active citation relationships. The Cell Death and Differentiation had the highest number of citations (N = 5,160) despite barely six publications, whereas Frontiers in Immunology was the most prominent node with the highest number of publications (N = 111) and total link strength (N = 725) (**Figure 6B**; **Table 3**), demonstrating their significant impact in the field of NETs and tumors.

**Table 3:** The top 10 most prolific journals and highly co-cited journals.

Rank	Journal	Documents	Citations	H-index	IF (2023)/JCR division	Co-cite journal	Citations	IF (2023)/JCR division
1	FRONTIERS IN IMMUNOLOGY	111	3,678	30	5.7/Q1	BLOOD	4,674	21.0/Q1
2	INTERNATIONAL JOURNAL OF MOLECULAR SCIENCES	66	1,240	20	4.9/Q1	JOURNAL OF IMMUNOLOGY	3,405	3.6/Q2
3	FRONTIERS IN ONCOLOGY	32	269	9	3.5/Q2	FRONTIERS IN IMMUNOLOGY	3,112	5.7/Q1
4	CANCERS	30	746	14	4.5/Q2	PROCEEDINGS OF THE NATIONAL ACADEMY OF SCIENCES OF THE UNITED STATES OF AMERICA	2,894	9.4/Q1
5	SCIENTIFIC REPORTS	21	551	13	3.8/Q1	NATURE	2,528	50.5/Q1
6	CELLS	21	368	11	5.1/Q2	PLOS ONE	2,412	2.9/Q1
7	JOURNAL OF LEUKOCYTE BIOLOGY	14	1,023	10	3.6/Q2	SCIENCE	2,308	44.7/Q1
8	JOURNAL OF THROMBOSIS AND HAEMOSTASIS	14	780	10	5.5/Q1	CANCER RESEARCH	2,274	12.5/Q1
9	THROMBOSIS RESEARCH	13	490	9	3.7/Q1	NATURE MEDICINE	2,114	58.7/Q1
10	CANCER RESEARCH	11	1,069	10	12.5/Q1	JOURNAL OF CLINICAL INVESTIGATION	2,064	13.3/Q1

IF, Impact factor; JCR, Journal Citation Reports.

**Figure 6C** maps the journal co-citation network by selecting journals with at least 250 citations, to illustrate the co-citation relationship between the two journals. Furthermore, an analysis of the journals from which the references originated shows the contribution of each journal to the knowledge base of the field. **Figure 6C** shows the 82 included journals classified into four clusters, where the size of the nodes represents the number of citations. There is an analogous theme between journals of the same color. Blood is located in the center of the triangle map and shares a broad spectrum of co-citation relationships with many other journals (**Figure 6C**). Consistent with this, Blood had the highest co-citations and total link strength (co-citations = 4,674, total link strength = 409,817), followed by the Journal of Immunology (co-citations = 3,405, total link strength = 339,642) and Frontiers in Immunology (co-citations = 3,112, total link strength = 270,942), all of which have substantial contributions to NET publications in tumors (**Figure 6C**). Likewise, **Table 3** presents the top 10 co-cited journals, each cited over 2,000 times. It is worth noting that all top the 10 co-cited journals have fairly high academic rankings, and almost all of them are in Q1 and have an IF of 10 or higher in 2023, with the highest being Nature Medicine, which has an IF of 58.7.

A dual-map overlay of journals created by CiteSpace portrays the topic distribution of scientific journals and illustrates the primary citation connections between citing and cited journals, with the colored path from left to right depicting citation pathways. The labels close to the emitting region represent the respective disciplines, with each label centered around the cluster centroid of the corresponding journals. As shown in **Figure 6D**, citing journals were chiefly from Molecular, Biology, Immunology and Medicine, Medical, and Clinical, called research frontiers, whereas the cited journals were chiefly from Molecular, Biology, Genetics and Health, Nursing, and Medicine, called the knowledge base. The longer the vertical axis of the ellipse, the more papers the journal publishes; the longer the horizontal axis, the greater the number of authors. Through the dual-map overlay of journals, it can be intuitively conjectured that the hotspots and frontiers of NETs in tumor research will progressively focus on the fields of Molecular, Biology, and Immunology.

### 3.6 Document citation analysis

**Figure 7:**
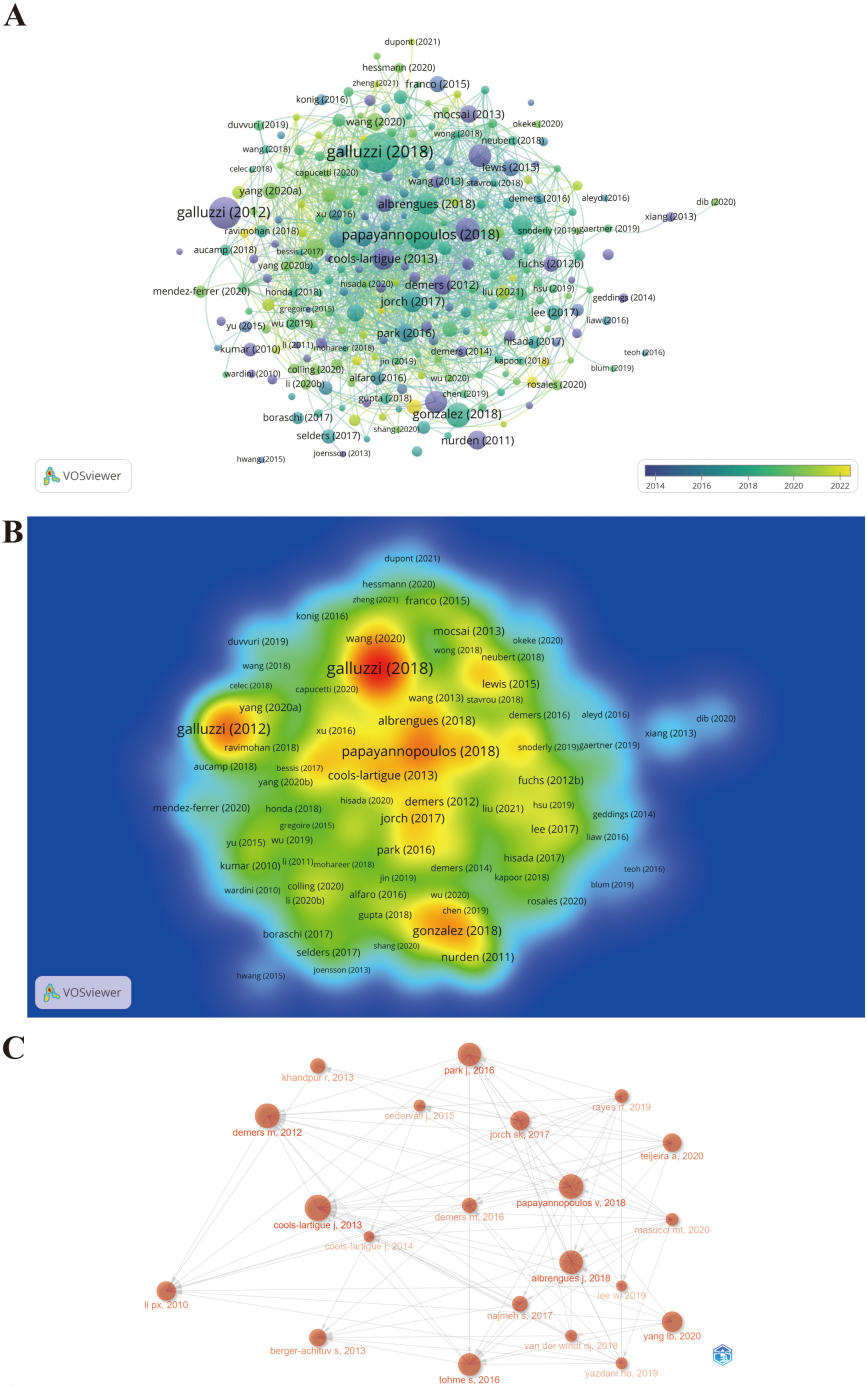
Maps of document citation analysis. **(A)** Overlay visualization map of document citation analysis. **(B)** Spectral density map of the document citations. **(C)** Association between the top 20 citation bursts.

The citation scores of 1,339 documents were analyzed using the Bibliometrix package, and the top 20 documents with the highest local citation score (LCS) and global citation score (GCS) are presented in **Table 4**. Specifically, for certain literature, LCS is the number of citations in the exported local database, which reflects the impact in this field, whereas GCS is the total number of citations in the Web of Science database, which reveals the impact in all fields. There are eight documents simultaneously listed in the top 20 of “Most Local Cited Documents” and “Most Global Cited Documents.” Wherein, the document with the highest LCS is the study published in the Journal of Clinical Investigation titled “Neutrophil extracellular traps sequester circulating tumor cells and promote metastasis” (LCS = 307), when the area just emerged. The possible reason why this incipient document has a profound impact on NETs and tumors is that the article is the first to reveal NET as a fully novel aspect of the basic biological characteristics of neutrophils contributing to tumor progression and metastasis and proposes that NET is a novel potential anti-tumor therapeutic target. Next, the document with the second highest LCS is the article published in Proceedings of the National Academy of Sciences titled “Cancers predispose neutrophils to release extracellular DNA traps that contribute to cancer-associated thrombosis” (LCS = 266), which reported that tumors induce a systemic environment priming neutrophils to release NETs, identified extracellular chromatin released through NET formation as a cause for cancer-associated thrombosis, and unveiled a target in an effort to decrease the incidence of thrombosis in cancer patients. Hence, these documents with high LCS provide a reference and theoretical basis for subsequent in-depth studies of the characteristics and biological functions of NET in tumors. Since then, scholars worldwide have conducted an increasing amount of research based on these classical studies. The document with the third highest LCS is the review subsequently published in Nature Reviews Immunology titled, “Neutrophil extracellular traps in immunity and disease” (LCS = 257). Likewise, the citation relationships of 249 documents with at least 50 citations are also portrayed in the overlay visualization map (**Figure 7A**), in which the size of the circle represents citations and the color denotes the publication year, while the impact of documents in the field is demonstrated in the density map in **Figure 7B**, in which the color depth represents citation intensity. In line with **Table 4**, Galluzzi et al. (72), Galluzzi et al. (73), and Papayannopoulos (74) are the three largest nodes (**Figure 7A**), accompanied by the brightest red color (**Figure 7B**). **Figure 7C** shows the citation relationship of the top 20 publications by local citation frequency, where the size of the circle represents citations, and the arrow denotes the citing direction. Cools-Lartigue et al. (75) and Demers et al. (76) received the most arrows as the two largest nodes (**Figure 7C**). Nevertheless, Papayannopoulos (74) does not receive the corresponding number of arrows as the third largest node in that the publications citing it are chiefly outside the top 20 publications.

**Table 4:** Citation score of documents (Top 20).

Rank	Most local cited document				Most global cited document	
	Title First author Journal Year	LCS	GCS	LC/GC Ratio (%)	Title First author Journal Year	GCS
1	Neutrophil extracellular traps sequester circulating tumor cells and promote metastasis Cools-Lartigue, Jonathan JOURNAL OF CLINICAL INVESTIGATION 2013	309	812	38.00	Molecular mechanisms of cell death: recommendations of the Nomenclature Committee on Cell Death 2018 Galluzzi, Lorenzo CELL DEATH DIFFERENTIATION 2018	3,208
2	Cancers predispose neutrophils to release extracellular DNA traps that contribute to cancer-associated thrombosis Demers, Melanie PROCEEDINGS OF THE NATIONAL ACADEMY OF SCIENCES 2012	269	607	44.19	Molecular definitions of cell death subroutines: recommendations of the Nomenclature Committee on Cell Death 2012 Galluzzi, Lorenzo CELL DEATH DIFFERENTIATION 2012	1,853
3	Neutrophil extracellular traps in immunity and disease Papayannopoulos, Venizelos NATURE REVIEWS IMMUNOLOGY 2018	263	1,505	17.26	Neutrophil extracellular traps in immunity and disease Papayannopoulos, Venizelos NATURE REVIEWS IMMUNOLOGY 2018	1,505
4	Neutrophil extracellular traps produced during inflammation awaken dormant cancer cells in mice Albrengues, Jean SCIENCE 2018	241	783	30.61	Roles of the immune system in cancer: from tumor initiation to metastatic progression Gonzalez, Hugo GENES & DEVELOPMENT 2018	1,037
5	Cancer cells induce metastasis-supporting neutrophil extracellular DNA traps Park, Juwon SCIENCE TRANSLATIONAL MEDICINE 2016	235	539	43.40	PAD4 is essential for antibacterial innate immunity mediated by neutrophil extracellular traps Li, Pingxin JOURNAL OF EXPERIMENTAL MEDICINE 2010	969
6	Neutrophil extracellular traps promote the development and progression of liver metastases after surgical stress Tohme, Samer CANCER RESEARCH 2016	217	428	50.71	NETs are a source of citrullinated autoantigens and stimulate inflammatory responses in rheumatoid arthritis Khandpur, Ritika SCIENCE TRANSLATIONAL MEDICINE 2013	875
7	DNA of neutrophil extracellular traps promotes cancer metastasis via CCDC25 Yang, Linbin NATURE 2020	175	414	42.16	Neutrophil extracellular traps sequester circulating tumor cells and promote metastasis Cools-Lartigue, Jonathan JOURNAL OF CLINICAL INVESTIGATION 2013	812
8	PAD4 is essential for antibacterial innate immunity mediated by neutrophil extracellular traps Li, Pingxin JOURNAL OF EXPERIMENTAL MEDICINE 2010	153	969	15.58	The multifaceted functions of neutrophils Mayadas, Tanya N. ANNUAL REVIEW OF PATHOLOGY-MECHANISMS OF DISEASE 2014	794
9	An emerging role for neutrophil extracellular traps in noninfectious disease Jorch, Selina K. NATURE MEDICINE 2017	151	729	20.64	Neutrophil extracellular traps produced during inflammation awaken dormant cancer cells in mice Albrengues, Jean SCIENCE 2018	783
10	CXCR1 and CXCR2 chemokine receptor agonists produced by tumors induce neutrophil extracellular traps that interfere with immune cytotoxicity Teijeira, Alvaro IMMUNITY 2020	140	316	44.16	An emerging role for neutrophil extracellular traps in noninfectious disease Jorch, Selina K. NATURE MEDICINE 2017	729
11	A proposed role for neutrophil extracellular traps in cancer immunoediting Berger-achituv, Sivan FRONTIERS IN IMMUNOLOGY 2013	125	188	66.31	Neutrophil: A Cell with Many Roles in Inflammation or Several Cell Types? Rosales, Carlos FRONTIERS IN PHYSIOLOGY 2018	641
12	Neutrophil extracellular traps sequester circulating tumor cells via β1-integrin mediated interactions Najmeh, Sara INTERNATIONAL JOURNAL OF CANCER 2017	105	184	56.91	Cancers predispose neutrophils to release extracellular DNA traps that contribute to cancer-associated thrombosis Demers, Melanie PROCEEDINGS OF THE NATIONAL ACADEMY OF SCIENCES 2012	607
13	NETs are a source of citrullinated autoantigens and stimulate inflammatory responses in rheumatoid arthritis Khandpur, Ritika SCIENCE TRANSLATIONAL MEDICINE 2013	101	875	11.60	Ulcerative colitis Kobayashi, Taku NATURE REVIEWS DISEASE PRIMERS 2020	596
14	Priming of neutrophils toward NETosis promotes tumor growth Demers, Melanie ONCOIMMUNOLOGY 2016	99	167	59.39	Platelets, inflammation and tissue regeneration Nurden, Alan T. THROMB HAEMOST 2011	548
15	Primary tumors induce neutrophil extracellular traps with targetable metastasis promoting effects Rayes, Roni F. JCI INSIGHT 2019	90	140	63.50	Cancer cells induce metastasis-supporting neutrophil extracellular DNA traps Park, Juwon SCIENCE TRANSLATIONAL MEDICINE 2016	539
16	The emerging role of neutrophil extracellular traps (NETs) in tumor progression and metastasis Masucci, Maria Teresa FRONTIERS IN IMMUNOLOGY 2020	86	198	43.68	Origins, structures, and functions of circulating DNA in oncology Thierry, A. R. CANCER AND METASTASIS REVIEWS 2016	494
17	Neutrophil extracellular traps accumulate in peripheral blood vessels and compromise organ function in tumor-bearing animals Cedervall, Jessica CANCER RESEARCH 2015	83	165	50.30	Diverse novel functions of neutrophils in immunity, inflammation, and beyond Mocsai, Attila JOURNAL OF EXPERIMENTAL MEDICINE 2013	488
18	Neutrophil extracellular traps drive mitochondrial homeostasis in tumors to augment growth Yazdani, Hamza O. CANCER RESEARCH 2019	83	113	74.77	Cytokine storm and leukocyte changes in mild versus severe SARS-CoV-2 infection: Review of 3939 COVID-19 patients in China and emerging pathogenesis and therapy concepts Wang, Jin JOURNAL OF LEUKOCYTE BIOLOGY 2020	462
19	Neutrophil extracellular traps promote inflammation and development of hepatocellular carcinoma in nonalcoholic steatohepatitis Van Der Windt, Dirk J. HEPATOLOGY 2018	83	265	31.42	Inhibition of PAD4 activity is sufficient to disrupt mouse and human NET formation Lewis, Huw D. NATURE CHEMICAL BIOLOGY 2015	440
20	Neutrophil extracellular traps in cancer progression Cools-Lartigue, Jonathan CELLULAR AND MOLECULAR LIFE SCIENCES 2014	82	164	50.63	Neutrophil Extracellular Traps and Its Implications in Inflammation: An Overview Delgado-rizo, Vidal FRONTIERS IN IMMUNOLOGY 2017	429

LCS, Local citation score; GCS, Global citation score.

### 3.7 Co-cited reference analysis

**Figure 8:**
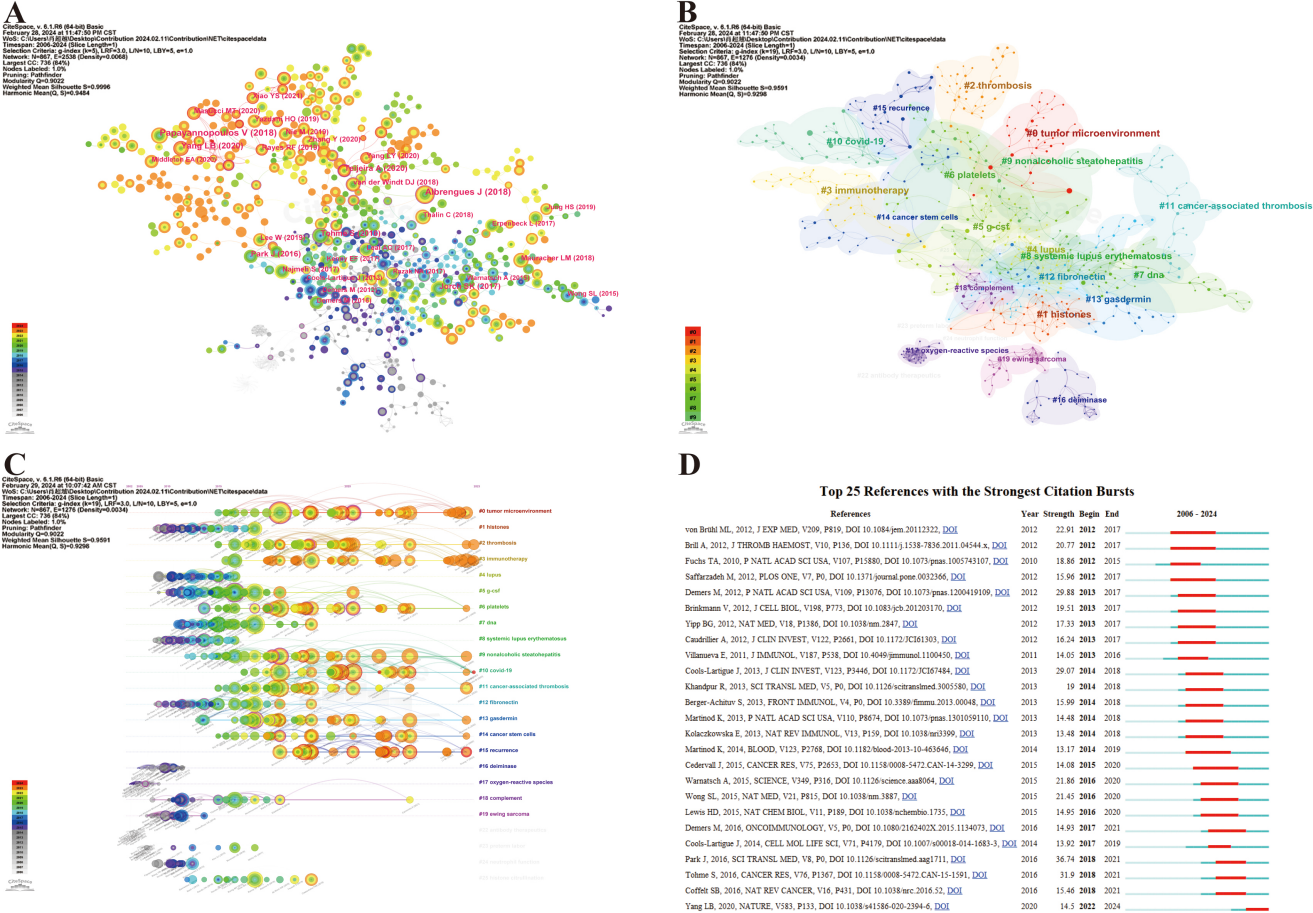
Maps of document citation analysis. **(A)** Co-citation analysis of references. **(B)** Clustering network analysis of co-cited references. **(C)** Timeline of the co-cited reference analysis. **(D)** Top 25 references with the strongest citation bursts.

Co-citation analysis can help us classify references and understand the current research field’s main components, as well as their evolution and development. We utilized CiteSpace to visualize the reference co-citation network, as depicted in **Figure 8A**, where 867 references with certain influences were extracted using the g-index, which allowed the identification of homogeneous clusters of references that were co-cited more frequently in the research. The labels show the first author with the 30 most-cited references and the year. Larger labels are assigned to authors based on the number of citations, and the links between nodes represent co-citation relationships. The size of the peripheral node (purple circles) represents intermediate centricity (in terms of numerical value). Additionally, considering that the 10 most co-cited references are also of interest to most oncologist researchers who focus on NETs, they are listed in **Table 5**.

**Table 5:** The top 10 co-cited references.

Rank	Co-cited reference	Citations	Centrality	First author	Year	Type	Journal	IF(2023)/JCR division
1	Neutrophil extracellular traps in immunity and disease	260	0	Papayannopoulos, Venizelos	2018	Review	NATURE REVIEWS IMMUNOLOGY	67.7/Q1
2	Neutrophil extracellular traps produced during inflammation awaken dormant cancer cells in mice	235	0.18	Albrengues, Jean	2018	Article	SCIENCE	44.7/Q1
3	DNA of neutrophil extracellular traps promotes cancer metastasis via CCDC25	175	0.01	Yang, Linbin	2020	Article	NATURE	50.5/Q1
4	CXCR1 and CXCR2 chemokine receptor agonists produced by tumors induce neutrophil extracellular traps that interfere with immune cytotoxicity	140	0.26	Teijeira, Alvaro	2020	Article	IMMUNITY	25.5/Q1
5	Cancer cells induce metastasis-supporting neutrophil extracellular DNA traps	134	0.10	Park, Juwon	2016	Article	SCIENCE TRANSLATIONAL MEDICINE	25.5/Q1
6	Neutrophil extracellular traps promote the development and progression of liver metastases after surgical stress	132	0.22	Tohme, Samer	2016	Article	CANCER RESEARCH	12.5/Q1
7	An emerging role for neutrophil extracellular traps in noninfectious disease	132	0	Jorch, Selina K.	2017	Review	NATURE MEDICINE	58.7/Q1
8	Primary tumors induce neutrophil extracellular traps with targetable metastasis promoting effects	89	0.06	Rayes, Roni F.	2019	Article	JCI INSIGHT	6.3/Q1
9	The emerging role of neutrophil extracellular traps (NETs) in tumor progression and metastasis	86	0.02	Masucci, Maria Teresa	2020	Review	FRONTIERS IN IMMUNOLOGY	5.7/Q1
10	Neutrophil extracellular traps promote inflammation and development of hepatocellular carcinoma in nonalcoholic steatohepatitis	82	0.12	Van Der Windt, DJ	2018	Article	HEPATOLOGY	12.9/Q1

IF, Impact factor; JCR, Journal Citation Reports.

The noun phrases are extracted from the keywords of reference and constitute the cluster labels by using the “All in one: clustering, optimizing layout and style.” Therefore, the reference co-citation analysis was divided into 95 divergent clusters, in which the largest 24 clusters were thoroughly examined. The modularity Q and the mean silhouette S were higher than 0.9, indicating a significant clustering effect and a highly credible network (**Figure 8B**). The clusters were numbered in ascending order, starting from 0, and the numbers represent the number of studies contained within each cluster, with smaller numbers indicating more studies within the corresponding cluster. After understanding the composition of the research content, its temporal evolution process naturally needs to be answered. Accordingly, we created a timeline view of the first 24 clusters by exhibiting nodes symbolizing individual references, illustrating the time span and research progress of the developmental evolution of distinct research topics (**Figure 8C**). Nodes appear on timelines in diverse positions and colors based on the time of reference publication. Nodes on the left represent earlier references, whereas those on the right represent more recent references. Nodes positioned along the same line represent a cluster, identified by label # on the right side; the length of the horizontal line and the fore and aft ends are the durations for that category. Overall, most of the highly co-cited references and the rise in co-citations in this field emerged in 2015, and many of the co-cited references continue to be widely cited to this day, denoting that NETs and tumors remain a prominent research topic that has been making rapid advancements and a vast array of results. Specifically, the largest cluster was named #0 tumor microenvironment with a long time span, reflecting most studies on NETs and tumors within this cluster, thus highlighting the significance of the tumor microenvironment in this research area. Furthermore, it can be observed that the hotspot has already shifted from the initial topic of study in this field at the line’s left end (e.g., #1 histones, #12 fibronectin, #16 deiminase, #17 oxygen-reactive species, and #19 Ewing sarcoma), whereas clusters #0 tumor microenvironment, #2 thrombosis, #3 immunotherapy, #6 platelets, #13 gasdermin, #14 cancer stem cells, #15 recurrence, etc., are located at the line’s rightmost end, representing the current new hotspots in this research (**Figure 8C**). In particular, hotspots #0 tumor microenvironment and #3 immunotherapy generated multiple highly co-cited references over the past year or two, demonstrating that they have been gaining significant attention.

Additionally, reference citation bursts were applied to highlight the popularity and significance of specific references in the NET field over time, and the top 25 references with the strongest citation bursts sorted by the beginning year of the burst are depicted in **Figure 8D**. The green line represents the period from 2006 to 2024, whereas the red line demonstrates the duration of each citation burst during which the references were cited the most, which may reveal that a novel research hotspot may be emerging in this field. According to **Figure 8D**, co-cited references with high citation bursts predominantly center on high-quality literature with a high IF in which there are five reviews in total. The onset of the reference citation burst can be traced back to 2012, whereas the latest occurred in 2022, attributed to Yang et al. (77), which is still undergoing a citation explosion phase even to this day and has the current emergence of strong citation references. Notably, the article entitled Park J, 2016, SCI TRANSL MED, V8, P0 experienced the strongest burst (Strength = 36.74).

### 3.8 Keyword and trend topic analysis

**Figure 9:**
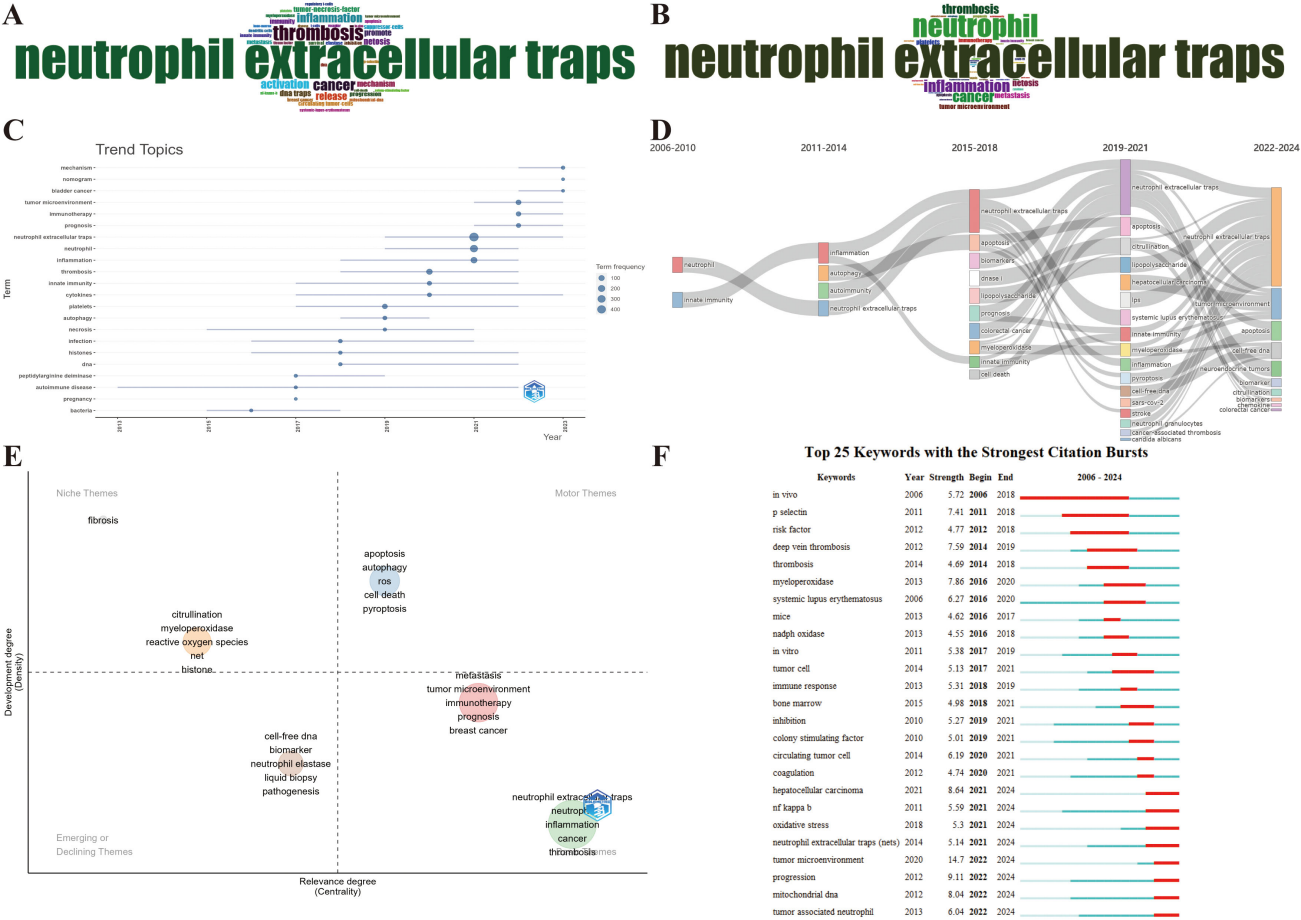
Maps of keyword and trend topic analyses. **(A)** Word cloud of the top 40 Keywords Plus. **(B)** Word cloud of the top 40 Author keywords. **(C)** Keyword-based research theme trend map. **(D)** Alluvial diagram of thematic evolution. **(E)** Strategic diagram of the subperiod. **(F)** Top 25 keywords with the strongest citation bursts.

As a concise overview of the article’s topic, keywords can be employed to analyze the current research status, hotspots, and future directions of NETs and tumors. First, the top 40 Keywords Plus and Author keywords were examined using a word cloud. In **Figure 9A**, the keyword “neutrophil extracellular traps” is in the center of the cloud map, followed by thrombosis and cancer. **Figure 9B** shows that “neutrophil extracellular traps” were the most prominent, followed by neutrophil and inflammation are close behind. Apparently, Keywords Plus is broader than Author keywords. Notably, as shown in **Table 6**, neutrophil extracellular traps (N = 930), DNA traps (N = 92), and platelets (N = 91) were the most commonly involved molecules or cellular products. Inflammation (N = 271), thrombosis (N = 206), and NETosis (N = 168) appeared more frequently in the pathological processes. Breast cancer (N = 81), colorectal cancer (N = 46), and lung cancer (N = 42) received the most attention.

**Table 6:** The top 10 molecule/cellular products, pathological processes and diseases related to NETs and tumor.

Rank	Molecule/Cellular product	Counts	Pathological process	Counts	Disease	Counts
1	Neutrophil extracellular traps	930	Inflammation	271	Cancer	292
2	DNA traps	92	Thrombosis	206	Breast cancer	81
3	Platelets	91	NETosis	168	Colorectal cancer	46
4	Tumor-necrosis-factor	82	Activation	133	Lung cancer	42
5	Elastase	58	Metastasis	132	Hepatocellular Carcinoma	41
6	Myeloperoxidase	57	Release	113	Pancreatic cancer	35
7	Tissue factor	56	Promote	88	Gastric cancer	20
8	DNA	56	Apoptosis	75	Melanoma	16
9	Mitochondrial-DNA	50	Progression	75	Prostate cancer	12
10	NF-kappa-B	43	Survival	54	Polycythemia-vera	8

To conduct a comprehensive analysis of the research trends, we employed the Bibliometrix package in R to visualize the annual primary keyword evolution, which was carried out with specific parameters in place, encompassing a designated timeframe from 2006 to 2024, a minimum word frequency threshold of 5, and a maximum of three words per year after amalgamating the synonymous keywords and expunging meaningless keywords. **Figure 9C** presents each topic as a line whose span represents duration, with the circle denoting the most prevalent year for that particular topic. A larger circle indicates that a theme appears more frequently. Before 2017, the research hotspots were relatively homogeneous, merely focusing on few keywords in the bottom left-hand corner of **Figure 9C**, such as bacteria, autoimmune disease, and necrosis. However, research hotspots have witnessed a sudden surge since 2017. Mechanism, nomogram, bladder cancer, tumor microenvironment, immunotherapy, prognosis, and cytokines continue to be subjects of intense research interest. The emergence of new keywords in the top-left corner of **Figure 9C**, such as mechanism, bladder cancer, tumor microenvironment, immunotherapy, prognosis, and nomogram, has gained prominence over the last 2 years, reflecting a discernible shift in the emphasis and orientation of future research endeavors. Furthermore, the alluvial diagram using the Bibliometrix package reveals the changes and internal connections of keywords in different periods. As depicted in **Figure 9D**, the research topics have shown a sudden surge since 2015–2018, in which some studies have been enduring in recent years, such as apoptosis, citrullination, and cell-free DNA. Notably, the tumor microenvironment has evolved into the most salient emergence since 2022, which aligns with the outcomes of the aforementioned annual trend chart of keyword changes. Additionally, a strategic diagram of the sub-period by the Bibliometrix package displays the development trend and maturity level of the keywords. As shown in **Figure 9E**, multifarious cell deaths (e.g., apoptosis, autophagy, and pyroptosis) are located in the Motor Themes quadrant, indicating that the above keywords are the core theme with high maturity. Citrullination, myeloperoxidase, reactive oxygen species, and histone are located in the Niche Themes quadrant, indicating that they are well-developed but banal. Cell-free DNA, biomarker, neutrophil elastase, liquid biopsy, etc., are located in the Emerging or Declining Themes quadrant, suggesting that the above keywords are peripheral themes and have not developed well. On the flipside, tumor microenvironment, immunotherapy, metastasis, inflammation, thrombosis, etc., are located in the Basic Themes quadrant, demonstrating that the above keywords are significant, but the current research is not sufficient, so the above topics may become research hotspots or future development trends.

Another meaningful indicator of advancements in research frontiers, fields of intense activity, and emerging patterns over time is the magnitude of the bursts seen in the keywords. **Figure 9F** presents the citation bursts of the top 25 keywords, where the time interval and duration of the citation bursts are marked in blue and red, respectively. The onset of a keyword citation burst can be traced back to *in vivo* (2006–2018), which also occupies the longest time span of a citation burst. Significantly, the citation burst time of terms in the bottom right-hand corner of **Figure 9F**, such as tumor microenvironment (2022–2024), tumor-associated neutrophil (2022–2024), mitochondrial DNA (2022–2024), NF-kappa-B (2021–2024), and oxidative stress (2021–2024) was sustained in 2024, with ongoing signs manifesting a notable surge in scholarly interest within specific fields of research. In particular, wherein tumor microenvironment has also been reaping the strongest citation bursts (strength = 14.7), which further highlights the prevalence and significance of the tumor microenvironment in the current field.

Notably, these findings align with the outcomes of the aforementioned reference co-citation analysis. Collectively, these findings identified research hotspots and illuminated trends in NETs and tumors, revealing a pronounced emphasis on molecular mechanisms in contemporary research.

## 4 Discussion

We applied five bibliometric tools to conduct a bibliometric analysis and visualization of 1,339 related publications extracted from WoSCC from 2006 to 1 February 2024, which systematically appraised the current research status and future research interests in the link between NETs and tumors. To the best of our knowledge, this is the first bibliometric review, summary, and outlook of NETs in the tumor field, which will be discussed in detail in the following sections, providing a comprehensive overview of our findings.

### 4.1 General information study

In the early 21st century, scholars focusing on the role of NETs in tumors have gradually emerged. Initially, this adventurous perspective failed to draw much attention; therefore, the annual number of relevant publications did not exceed 10. With researchers’ realization of the promising potential of NETs as novel targets for anti-tumor therapy, this field soon became hot and has been further excavated in the past decade. We speculated that the number of publications pertinent to NETs would persistently boost tumor research.

Regarding geographical distribution, there were merely 70 countries/regions encompassed in our study, and less than half of the countries/regions published more than 10. Intriguingly, the United States followed China in terms of the number of publications, but has continued to dominate the field as a global cooperation center with overwhelming citation counts, partially owing to their long-standing foundation in the biomedical field and indispensable funding and ample scientific institutions and investigators in this field, which evinces that the United States had conducted in-depth research in this field and the potentially greater leverage in their articles. For highly regionalized cooperation, extending international and cross-boundary collaboration is imperative, especially in developing countries. Furthermore, the role of fiscal support and research grants in redounding scientific output cannot be assessed. Many countries may profit from incremental investments and policy encouragement for research, which expedites their emergence as considerable contributors in this field.

In terms of authorship, our analysis also discloses a disparity between productivity and impact, with some highly productive authors not having a high impact, and vice versa. Specifically, only Wagner and Kaplan are concurrently in the lists of both the top 10 most prolific authors and the top 10 most cited authors. For instance, Tang and Wang had high citation counts but did not publish a large number of papers, reflecting that they sustained stringent quality control over their publications.

Academic journals focus more on journals with more publications and citations and higher IF. Frontiers in Immunology has published the most papers on NETs and tumors, indicating that more meaningful findings are likely to be published in this journal. Additionally, Blood, Journal of Immunology, and Frontiers in Immunology were co-cited more than 3,000 times, with Blood possessing the most co-citations, which is indicative of the higher quality and reference value of this journal in this field. Except for the Journal of Immunology, all the co-cited journals in **Table 3** are located in Q1, demonstrating the significance of NETs in future tumor research.

### 4.2 Emerging trends, hotspots, and frontiers

The performance of reference co-citation networks and keyword analysis not only maps the outline of historical and current research structures but also provides a foresight into the future orientation, encompassing but not confined to the tumor microenvironment, immunotherapy, and so on. These findings allow for the identification of emerging trends and research hotspots in the link between NETs and tumors. Below, we elucidate their far-reaching meaning for this realm and the latent implications for future directions.

#### 4.2.1 Crosstalk between NETs and TME

Recently, mounting evidence has demonstrated that uncontrolled or inordinate formation of NETs plays multifarious fundamental roles in tumors by interplaying the components within the TME, which represents an intricate ecosystem composed of tumor and mass non-tumor cells (e.g., diversified immunocyte subpopulations, cancer-associated fibroblasts (CAFs), endothelial cells, pericytes, and additional tissue-resident cell subpopulations) embedded within the significantly altered extracellular matrix. As an emerging research focal point, the identified repertoire of crosstalk between NETs and the TME has been expounded. This information is pivotal in determining the precise molecular mechanism of NET-cell contact for targeted therapy and intervention.

##### 4.2.1.1 Interplay between NETs and tumor Cells

There is growing evidence from ligand/receptor pair investigations that NETs can be induced by tumor cell-associated factors (e.g., IL-8, G-CSF, GROα/β, and CXCR1/2 chemokine receptor agonists) and chiefly facilitate tumor cell proliferation, adhesion, and metastasis within the TME (13, 14). For instance, tumor cell-released autophagosomes (TRAPs) induce NET formation by using HMGB1 and activating the TLR4-Myd88-ERK/p38 pathway, culminating in breast cancer pulmonary metastasis (15). Hepatocellular carcinoma cells facilitate neutrophil recruitment and NET formation in the metastatic microenvironment by decreasing histidine-rich glycoprotein secretion, culminating in lung tumor metastasis (16). On the other hand, NETs directly or NET-derived factors interact with the receptors on tumor cells and thus reshape their functions. The most studied examples were the NET-derived factors and TLR family in tumor cells. For instance, neutrophil elastase (NE), a protein coated in the decondensed DNA within NET, augments invasion and metastasis through NLRP3-mediated suppression of pyroptosis in oral squamous cell carcinoma (17). NE also directly activates the TLR4 pathway in tumor cells and thereby upregulates peroxisome proliferator-activated receptor gamma coactivator 1-alpha, culminating in mitochondrial homeostasis and colorectal cancer progression. The secretory protein high mobility group box 1 (HMGB1) augments NET formation via a TLR4-dependent manner and consequently contributes to radiotherapy resistance of bladder cancer, and blocking NETs or HMGB1 can ameliorate radiation response (18). Additionally, Interleukin-8 secreted by lymphoma cells binds to C-X-C Motif Chemokine Receptor 2 on the neutrophil surface and induces NET formation, which in turn activates TLR9 on lymphoma cells and thereby activates nuclear factor kappa B (NF-κB), STAT3, and p38 downstream pathways, culminating in the progression of diffuse large B-cell lymphoma (19). NETs interact with TLR9 to reduce Merlin phosphorylation, thereby inducing tumor cell ferroptosis resistance and culminating in triple-negative breast cancer progression (20). NETs can be released more by neutrophils derived from metastatic hepatocellular carcinoma than by those in healthy adults, thereby elevating the invasion capability of trapped tumor cells by activating the TLR4/9 receptor, phosphorylating P65, and overexpressing cyclooxygenase-2 (21). In addition, as a transmembrane protein in breast cancer cells, CCDC25 can directly interact with the NET–DNA complex, which facilitates the motility and metastasis of tumor cells via the activation of downstream pathways encompassing integrin-linked kinase and β-parvin (22). Likewise, NET–DNA interacts with αV integrin in cholangiocarcinoma cells, mediating tumor proliferation, metastasis, and angiogenesis by activating the NF-κB pathway (23). NETs upregulate the expression of α5β1 integrin on colorectal cancer cells, enhancing tumor adhesion, proliferation, and migration (24).

It is also essential to acknowledge the controversial dual effects of NETs on tumor cells. NETs can elicit cytotoxicity and hinder the migration and proliferation of human melanoma and bladder cancer cells *in vitro* (25). Chemotherapy-induced NETs curb tumor progression in murine models of colorectal cancer (26). Nevertheless, the factors that dictate whether NETs are pro- or anti-tumor are nebulous. Altogether, although very few publications now illustrate the *in vitro* antineoplastic effect of NETs, their definite role and molecular mechanism deserve further investigation, especially for their ligand–receptor pairs. Furthermore, targeting ligand–receptor pairing or specific kinases rather than neutrophils or tumor cells could be a promising strategy for antineoplastic therapy.

##### 4.2.1.2 Interplay between NETs and immunocytes

The convoluted interaction between NETs and immunocytes may critically involve the establishment of an immunosuppressive niche that promotes tumor evasion and therapy resistance. CD8^+^ T-cell infiltration is inversely associated with NET density in human non-small cell lung and bladder cancers (27). The motility of CD8^+^ T cells migrating across the transwell is directly abated by NETs *in vitro* (13). CD8^+^ T cells within the NET-rich TME are characterized by a functional and metabolically exhausted phenotype, expressing multiple exhaustion markers (e.g., PD-1, LAG-3, and TIM-3). Consistent with the alterations within the NET-rich TME, NETs skewed T cells to exhaustive differentiation by co-culturing *in vitro*, which can be reversed by NET blockade (28, 29). PD-L1 carried by TRAP-induced NETs impedes T-cell function *in vitro* and *in vivo*, thereby forming premetastatic lung niches (15). The tumor-derived cytokine Chi3l1 induces NETs and promotes CD8^+^ T-cell exclusion in triple-negative breast cancer (30). Significantly, the NET–DNA structure acts as a physical barrier that shields tumor cells from cytotoxicity evoked by CD8^+^ T and natural killer (NK) cells by wrapping and coating tumor cells, as well as obstructing their mutual contact (13). NET–DNA can bind transmembrane and coiled-coil domains 6 on CD8^+^ T cells to emasculate antineoplastic immunity, thus accelerating the progression of hepatocellular carcinoma (31). On the other hand, CD4^+^ T cell exhausted phenotype differentiation and Foxp3^+^ regulatory T cell (Treg) density are positively correlated with NETs (28, 32). NETs foster crosstalk between innate and adaptive immunity during carcinogenesis during non-alcoholic steatohepatitis (NASH) progression by promoting Treg differentiation via metabolic reprogramming of naïve CD4^+^ T cells (32). Mechanistically, NETs are abundant in NASH livers, which directly contact naïve CD4^+^ T cells mostly via TLR4 and thus prompt mitochondrial oxidative phosphorylation (OXPHOS), culminating in Treg differentiation. OXPHOS inhibitors reverse NET-associated Treg differentiation (32). Furthermore, collagen-induced DDR1 upregulates CXCL5 to enhance NET generation, thereby enhancing Treg differentiation and infiltration in breast cancer (33). NETs upregulate CD73 by activating Notch2 mediated NF-κB pathway, facilitating Treg infiltration to induce immune evasion in hepatocellular carcinoma (34). In addition, IL-17 secreted by γδ T cells can foster NET formation and may further repress CD8^+^ T-cell recruitment to tumors (35).

Moreover, the acidic TME and NETs notably hinder the efficacy of NK cell infusion, but dual pH-responsive hydrogels with cancer acidity neutralizer and NETs lyase Deoxyribonuclease I combined with NK cell infusion can effectively counteract postoperative relapse of hepatocellular carcinoma (36). Colorectal cancer region-specific CD16^+^ neutrophils competitively repress the cholesterol intake of NK cells to block their cytotoxicity but also release NETs to induce NK cell death. CD16-knockout reverses neutrophil carcinogenesis and NK cell cytotoxicity (37). Complement C5a induces the surface expression of HMGB1 receptors TLR4 and RAGE in polymorphonuclear-granulocytic-myeloid-derived suppressor cells (MDSCs), thereby favoring tumor cell invasion based on NET formation. Inhibition of C5a, C5a receptor, or NETosis curtails tumor cell dissemination and metastasis (38, 39). Knockdown of the triggering receptor expressed on myeloid cell 1 obstructs NET-mediated M2 macrophage polarization, thereby hampering gastric cancer progression (40).

Like most other immunocytes, neutrophils can adopt either pro- or anti-tumorigenic immunoregulatory functions, and this is also the case for NETs. For instance, injecting NET proteins into subcutaneous tumors is responsible for the recruitment of more T cells and monocytes and macrophages, cancer cell death, and reduced tumor size in bladder tumor-bearing murine model (41). Additionally, the interplay between NETs and dendritic cells (DCs) allows DCs activation and maturation toward the presentation of NET-associated antigens. Leukemic cells can release prototypical mutant nucleophosmin (NPMc^+^), which translocates from the nucleolus to the cytoplasm and localizes onto NET threads. Vaccination with DCs loaded with NPMc^+^ NETs can reduce myeloproliferation in transgenic murine model, favoring the development of antibodies against mutant NPMc and triggering the CD8^+^ T-cell response (42). Nevertheless, the crosstalk between NETs and B cells in tumors is poorly understood and deserves further exploration.

##### 4.2.1.3 Interplay Between NETs and Others

The interaction between neutrophils and platelets promotes NET generation, which provides a scaffold to platelets and triggers platelet activation and aggregation by their complex components, thereby forming a positive feedback loop. Specifically, administration of anti-P-selectin and PSGL-1 antibodies to abolish the interplay between platelets and neutrophils could potently suppress NET generation in the plasma of glioma patients (43). Moreover, platelets from colorectal cancer patients trigger neutrophils to release NETs, which can be abrogated by the absence of the pro-inflammatory molecule platelet-derived HMGB1. Simultaneously, platelets serve as carriers of tumor-derived exosomes and contribute to NET formation. In contrast, NETs function in procoagulation by providing a scaffold for platelets, red blood cells, extracellular vesicles, and pro-coagulant molecules (44). NETs skew platelets to a procoagulant phenotype and elicit platelet activation and aggregation by overexpressing phosphatidylserine and P-selectin on their membranes (43, 45). Furthermore, DNase I, an enzyme that degrades NETs, dampens platelet aggregation, but some platelets still adhere to glass slides in that the most copious protein histone in NETs or NE is sufficient to mediate platelet aggregation (45).

Proteases (e.g., matrix metalloproteinase 9 and NE) in NETs can dissolve the extracellular matrix, thereby releasing vascular endothelial growth factor to enhance tumor invasion and angiogenesis. NETs ensnare tumor cells that adhere them to vascular walls through the von Willebrand factor, thereby disrupting the junctions between endothelial cells and enhancing the blood vessels’ permeability. This facilitates the passage of tumor cells through vessel walls to distant tissues and the establishment of micrometastatic foci (46). CAFs, an essential component of the tumor stroma, play multifaceted roles within the TME. CAF-secreted Amyloid β evokes tumor-associated NET formation through CD11b in an ROS-dependent mechanism both within the TME and at systemic levels in the blood and bone marrow (47). Fibroblast growth factor 19-induced inflammatory CAFs facilitate neutrophil infiltration and NET formation in liver metastatic niches by generating complement C5a and IL-1β, culminating in liver colonization of colorectal cancer cells (48). Reciprocally, NETs facilitate liver micrometastasis in pancreatic ductal adenocarcinoma (PDAC) by activating CAFs (49). There are more visceral adipocytes in the pancreas of obese mice, which promotes neutrophil recruitment and NET formation, thereby inducing pancreatic carcinogenesis (50).

#### 4.2.2 Targeting NETs for synergizing immunotherapy

Recently, there has been a growing interest in the mechanism of the tumor immunosuppressive microenvironment, especially after the clinical application of immune checkpoint inhibitors (ICIs) with unparalleled efficacies. Approximately 60%–80% of patients remain unresponsive or resistant to ICIs as a single agent. When coupled with the apparent toxicity and high cost of existing immunotherapies, accentuation of the response rate and mitigation of adverse reactions is a pivotal issue that clinicians urgently address (51). Given the overwhelming evidence supporting the notion that NETs exert pro-tumorigenic immunoregulatory functions in penetrative crosstalk with components within the TME, it is now increasingly accepted that NET inhibition maximizes the antineoplastic efficacy of ICIs in the future (52–55).

NETosis is a typical process of NET formation and has recently emerged as a promising target for augmenting immunotherapy (56, 57). For example, a Tandem-locked NETosis Reporter 1 can activate fluorescence signals only in the presence of both NE and cathepsin G to noninvasively monitor NETosis, providing prognostic assessment for immunotherapy (58). The melanoma cell-implanted murine model showed tumor regression with ICIs, and NET increment could be involved in immunotherapy-induced adverse reactions (59). Inhibition of peptidylarginine deiminase 4 (PAD4) can diminish circulating NETs and tumor-mediated NET generation (60). NET deficiency in a murine model deficient in PAD4 delayed tumor progression and extended survival (13, 35, 60). Specifically, CD8^+^ T-cell recruitment and its mediated cytotoxicity are responsible for the arrest of tumor progression (13, 35). Consequently, ICIs are endowed with more efficacious immune-mediated tumor regression in synergy with PAD4 inhibition (13, 35). Similarly, DNase I powerfully dampens resistance to ICIs and curbs tumor growth and immune escape in that DNase I-induced NETs degradation increases CD8^+^ T cell infiltration and cytotoxicity but decreases Treg infiltration and tumor metastasis (15, 34). CXCR1/2 are potent mediators of neutrophil chemotactic recruitment and NETosis (13, 61), whereas IL-8 is a significant mediator of CXCR1/2-mediated human neutrophil chemotaxis (27, 61). Circulating IL-8, as well as circulating and tumor-infiltrating neutrophils, are directly associated with poorer responses to ICIs (13, 62), and IL-8 expression and NET deposition are inversely associated with CD8^+^ T cell number in bladder cancer, metastatic melanoma, and non-small cell lung cancer (27). Therefore, blocking both neutrophil recruitment and NETosis via IL-8 or its receptor (CXCR1/2) inhibition in synergy with ICIs aggravates immune-mediated cytotoxicity to tumors (13, 62), and clinical trials alone or in conjunction with anti-PD-L1 antibodies are ongoing. Additionally, IL-17 can recruit neutrophils, evoke NETs, and exclude cytotoxic CD8^+^ T cell subpopulations from tumors, in which evoked NETs induce resistance to ICIs in PDAC (35). Thus, blocking IL-17 can enhance sensitivity to ICIs, and repressing neutrophils or Padi4-dependent NETosis can phenocopy IL-17 neutralization (35). In addition, NETs released by spontaneously activated neutrophils from patients with PDAC generate a microdomain where cathepsin S (CTSS) cleaves human arginase 1 (hARG1) into divergent molecular forms with elevated enzymatic bioactivity at physiological pH (63). NET-pertinent hARG1 represses CD8^+^ T cells whose proliferation is restored via administration of hARG1-specific monoclonal antibodies or preventing CTSS-based cleavage, while small-molecule blockades are invalid. ARG1 inhibition in synergy with ICIs can revitalize CD8^+^ T cell functions in PDAC tumors. Moreover, hARG1-specific monoclonal antibodies enhance adoptively transferred tumor-specific CD8^+^ T-cell frequency within the TME and augment ICI efficacy. Gsk3a induces neutrophil recruitment and NET formation, resulting in inhibition of cytotoxic T lymphocytes (CTLs) (64). Targeting Gsk3a can intensify CTL function and increase the efficacy of anti-PD-1 antibody.

Notably, systemically biodistributed NET inhibitors have safety issues that could undermine host defenses targeting infections. With the increasing penetration of emerging nanotechnology in the biomedical field, tumor-specific delivery and metastatic niche targeting effects symbolize prospective antineoplastic regimens for local inhibition of NETs (65–70). As expected, the use of nanoplatforms effectively ameliorated ICI efficacy in murine model with primary colorectal cancer (71). Collectively, current studies on NETs and immunotherapy are chiefly conducted *in vitro* or *in vivo*, while there is a lack of evidence for the effects of NETs on immunotherapy in humans. The study aimed to scrutinize the correlation between NET and immunotherapy efficacy and adverse reactions in clinical cohorts.

### 4.3 Limitations

This study has unavoidable limitations. First, given that the research is restricted to papers from WoSCC, certain relevant papers from other databases (e.g., PubMed, Embase, and Scopus) may have been missed. Next, it is possible that non-English high-quality papers are overlooked because the focus is merely on English papers, which would have introduced selection bias. Thus, the latest high-quality papers with a low citation rate may not sufficiently encapsulate their academic value and are not included in this study due to temporal restrictions. Moreover, owing to the inability of existing bibliometric tools to statistically analyze and visualize the journals of publication in country/institution/author/document citation/reference analysis, merely citing numbers of publications and citations without assessing the IF or quality of journals cannot fully reflect reality. Lastly, the bibliometric methods adopted here are restricted to metadata rather than a full-text analysis, so we may have missed key points that barely appear in the full articles, such as the author’s perspectives and outlook on the area. Although the aforementioned restrictions would not alter the findings of this study, future work should further consolidate the research foundation to encompass non-English papers and the latest notable studies.

## 5 Conclusions

A comprehensive bibliometric analysis was conducted to map the current landscape and knowledge structure of the link between NETs and tumors. According to a systematical analysis, NETs play a fundamental role in tumors. The number of publications about NETs and tumors has been steadily rising year by year, indicating that research is undergoing a vibrant and rapidly evolving era. Despite China’s maximum quantity of publications, the United States has continued to dominate the field as a global cooperation center with overwhelming citation counts. Frontiers in Immunology published the most publications, whereas Blood was the most co-cited journal. Wagner and Kaplan are the top 10 most prolific and cited authors. Insights into the link between NETs and tumors are increasing at an unprecedented speed, yet it remains much left to depict the NET-mediated molecular networks in tumor immunity. Our analysis prefigures the future trajectories of the field: tumor microenvironment and immunotherapy will likely be the focal points of future research. There is a growing recognition that NETs are attractive targets because of their multifaceted roles in tumor immunity, and perhaps the eventual remaining destination in the realm is to translate NET-targeted immunotherapies into clinical practice.

## Data Availability

The original contributions presented in the study are included in the article/supplementary material. Further inquiries can be directed to the corresponding author.
